# Estimating staffing requirements using workload indicators of staffing need at Braun District Hospital in Morobe Province, Papua New Guinea

**DOI:** 10.1186/s12960-021-00677-x

**Published:** 2022-01-28

**Authors:** Dixon Dimiri, Nelson Mek, Mary Therese Apini, Thelma Ali, Grace Turi Pumuye, Varage John Laka, Rosemary Jogo, Pamela Kari, Deki, Okech Mollent, Dapeng Luo, Anna Maalsen, Katu Yapi, Robin Madodo

**Affiliations:** 1 Health Workforce Standards and Accreditation Branch, National Department of Health, Port Moresby, Papua New Guinea; 2 World Health Organization, Port Moresby, Papua New Guinea; 3 Lutheran Health Services, Port Moresby, Morobe Province Papua New Guinea

**Keywords:** Clinical staff, Non-clinical staff, Workload indicators of staffing need

## Abstract

**Background:**

Papua New Guinea has seen some improvements in health indicators over the past years, but the pace of improvements is not as robust as expected. The Health Services Plan for Braun District Hospital redevelopment identified the importance of reflecting the hospital’s role in the broader health system, particularly in upgrading the services to service a bigger population. In August 2020, the hospital was upgraded from a health centre—level 3 to a district hospital level 4. The need for assessing human resources for health requirements for this level of care was thus necessary.

**Methods:**

The National Department of Health approved the use of the workload indicators of staffing need as the best tool to support in estimating staff requirements for the newly upgraded hospital. The focus was on clinical and non-clinical staff. Using already developed workload components and activity standards by the expert working groups for level 4 facilities, we visited the facility and collected data through interviews with the Lutheran Health Services representative, hospital management and staff. The technical task force reviewed daily registers, monthly reports and the data in the electronic national health information systems. The information collected was analysed using the workload indicators of staffing need software and interpreted.

**Results:**

There were staffing shortages among the clinical staff like the medical officers, nursing officers, health extension officers, pharmacists, radiology staff unit and in the laboratory staff. Shortages among the non-clinical staff were recorded by the cashiers, security officers, drivers and boat skippers. The results showed that the facility lacks a medical laboratory technologist, pharmacists and a medical imaging technologist. The community health workers in this facility are utilized in all the areas where shortages are registered to multitask.

**Conclusion:**

The results from this WISN study provide evidence for basing staffing decisions on. The WISN results from Braun District Hospital show that the facility requires a total of 33 inpatient nurses against the existing 21 inpatient nurses thus giving a staff gap of − 12 and a WISN ratio of 0.67. It is thus recommended that the hospital management prioritizes recruitment of nurses or if no resources, reassign one of the outpatient nurses to alleviate the pressure among the inpatient nurses or the extra theatre nurses to offer some services in the inpatient wards. WISN results can help managers make decisions such as change of health facility status from a health centre to a district hospital.

## Background

Papua New Guinea (PNG) has a government-funded health system throughout the country. It is supplemented by government-subsidized health services provided by various Christian missions such the Lutheran Health services. The churches are a major partner providing 50% of the health care services mostly in the rural areas [1]. The 2007 Provincial Health Authority Act [2, 3] enabled the provincial government to establish Provincial Health Authorities (PHAs) responsible for both primary and secondary health care in the provinces. This legislation streamlines the provision of health services at the provincial level and brings together the provincial health departments, hospitals, and district health services under one management board.

The Health Plan [4] was developed to strengthen primary health care (for all) and improve service delivery to the rural majority and the urban disadvantaged and ensure quality health care services are readily accessible and affordable for all. The health services are organized into six levels of care namely the village health posts, community health posts, health centres, district hospitals, provincial hospitals and finally the national referral hospital [5–7].

The NDoH, Medical Standards Division, Workforce Standards and Accreditation Branch (WSA) have the mandate of streamlining workforce standards for the country to enable achievement of the Universal Health Coverage (UHC) and improve health outcomes in the country towards achievement of national and global health commitments [8]. Despite achieving considerable success through the multi-sectoral approach in strengthening the human resources for health capacities, there still exist some gaps such as imbalances in the development and distribution of health workers. In addition to shortages of qualified health professionals in underserved areas, there are significant variations in health services and their quality which causes inequities in health outcomes [9].

With the support of WHO, the WSA branch is working closely with PHAs and health facilities at various levels to advance long term solutions to improve health workers numbers distribution and utilization to ensure quality care for all. The country has integrated the use of workload indicators of staffing need as the evidence-based method for adoption for health worker planning [10].

From the early 1960s, Braun was a health centre serving a small island community which has since grown and services have equally expanded. It is run by Lutheran Health Services (LHS) and located in Finschafen District, Morobe Province in Momase Region of PNG. The hospital status was upgraded from Level 3 to Level 4 in August 2020. The facility has a bed capacity of 120 beds and serves a greater population with a staff capacity of 56 both clinical and non-clinical staff. The upgrading of Braun District Hospital was deemed necessary to ease referral for the primary care facilities in Morobe Province and decongest the provincial and regional facilities.

The overall goal of this study was to determine staffing requirements based on workloads for improved health services in the new upgraded hospital. The results would guide the hospital management board and sponsors on optimum numbers of health workers required per cadre for improved quality health services using WISN.

## Methods

The WHO WISN methodology was introduced and approved for use in the country in 2017. The WSA team was trained as the Technical Task Force (TTF) to lead the process of WISN in the country. Expert working groups (EWGs) for all the cadres were trained and later develop the workload components for all the cadres and the corresponding activity standards for adoption in the country. The workload components were validated, approved and printed for use in the country. Available working time (AWT) for the facility for each of the cadres was established. Using cadre-specific workload components, the TTF visited Braun District Hospital to gather health service statistics for 2019 for use in the WISN software. The results generated from the software were analysed and interpreted. Staff interviews, management and LHS administration were also conducted to enable further understanding. Other sources of health service statistics were the daily registers and the partial health information systems.

### Setting of the study

The study was set in Braun District hospital in Finschafen District, Morobe Province in the wider region of Momase in Papua New Guinea (PNG). The study focused on all the staff of Braun District Hospital. It included both the clinical and non-clinical staff in all the service areas of the hospital.

### Sampling design, size and procedure

This was a case-specific analysis for Braun District Hospital. Both clinical and non-clinical staff were included in the study. The clinical staff included: medical officers, nursing officers, midwives, health extension officers, community health workers, ophthalmic assistant, dental therapist, medical laboratory assistants, X-ray assistant, physiotherapist and pharmacy assistants. The non-clinical staff included: the hospitals’ health administrator, statistician, accountant, accounts clerk, cashier, key board operator, electrician, plumber, drivers, boat skippers, security officers and the morgue attendant.

The TTF held an initial meeting with all the service areas managers in the hospital to orient them on the agenda of the week to avoid disruption of the hospital services. The daily registers submitted provided the activities conducted and the records of each of the services. The human resource manager from LHS provided all the administrative records that supported the WISN study such as staff numbers, information on staff absences mainly the annual, training and sick leaves. An overview of the HRH management practices implemented by the hospitals under LHS was highlighted. This information was critical in the establishment of AWT for each of the cadres and shift programmes in the facility. Further, information about the services offered in the facility, service areas and current staffing based on the workload components developed for each cadre was collected. The hospital statistician shared the monthly and annual statistics from the electronic National Health Information System (eNHIS) which had data for some key indicators and not all services as listed in the data collection sheet. Data verification and validation were conducted before being uploaded into the WISN software.

## Results

For each of the cadres, AWT was established as shown in Table 1. The AWT ranged from 1760 to 2000 h for year of study. Both the inpatient and outpatient nurses registered an AWT of 1880 h each while the medical officer had a total of 1960 h. The health extension officer registered a total of 1840 h as AWT. While the dental therapist, medical laboratory assistants and the X-ray assistants all recorded 2000 h as AWT. The physiotherapist had an AWT of 1760 h for the year and the midwives had an AWT of 1888 h for the year 2019. The non-clinical staff worked under 2 types of shifts arrangements. There were those who worked for 8 h per day for 5 days in a week and rested on all the public holidays like the accountant, health administrator, statistician, accounts clerk, cashier and the key board operator. Drivers, boat skippers, security officers worked for 24 h per day, 7 days a week throughout the year. Their posts had to be filled throughout.

**Table 1:** Available working time for all the staff at Braun District Hospital

Cadre	Working days per week	Working hours per day	Annual leave	Public holidays	Sick leave	Training days	Non-working days	Non-working weeks	Working weeks	Working days	No. of hours per year
Dental therapist	5	8		10			10	2	50	250	2000
Laboratory assistant	5	8		10			10	2	50	250	2000
Health extension officer	5	8	15	10		5	30	6	46	230	1840
Medical officer	5	8		10		5	15	3	49	245	1960
Midwives	5	8	14	10			24	4.8	47.2	236	1888
Outpatient nurses	5	8	15	10			25	5	47	235	1880
Ophthalmic assistants	5	8	23	10			33	6.6	45.4	227	1816
In patient nurses	5	8	15	10			25	5	47	235	1880
Theatre nurses	5	8	15	10			25	5	47	235	1880
Anaesthetic technical officer	5	8		10			10	2	50	250	2000
Physiotherapist	5	8	30	10			40	8	44	220	1760
Pharmacy assistant	5	8	5	10			15	3	49	245	1960
X-ray assistant	5	8		10			10	2	50	250	2000

The study also involved non-clinical health workers who work for 8 h for 5 days per week and rest on public holidays at 2080 h per year

Another set were non-clinical staff whose positions are filled throughout the year at 8736 h per year in shifts

Still on Table 1, the results show that the facility had no records on the sick leaves from all the cadres. On training, only the medical officer and the health extension officer had at least 5 days each for training while other cadres had no training throughout the year. As for the annual leave which is a statutory requirement for all the cadres, not all were able to take the annual leaves. They included the dental therapist, laboratory assistant, medical officer, anaesthetic technical officer and the X-ray assistant.

The workload components for each of the cadres and their corresponding activity/service standards for the three workload groups namely: health, support and additional activities were provided for each of the cadres. Table 2 provides the three workload groups, service standards and annual statistics collected in the facility for the 13 clinical cadres under study.

**Table 2:** Workload components and activity standards and annual statistics collected

Medical officers		
Workload components	Service standards	Annual-2019
Health service activities (Group 1 activities)		
Consultations	20 min per patient	5880
Admissions	30 min per patient	3920
Ward rounds	20 min per patient	3920
Minor surgeries	17 min per patient	6918
Intermediate surgeries	45 min per patient	2613
Major surgeries	259 min per patient	454
Medical procedures	30 min per patient	3920
Complex deliveries	60 min per patient	520
Referrals	15 min per patient	321
Assault and injuries	30 min per patient	1840
Death declaration	10 min per patient	35
Discharge	10 min per patient	5524
Re-attendances	15 min per patient	11,760
Support activities (Group 2 activities)		
Morning devotion	45 min per day	
Daily debriefs	20 min per day	
Clinical case presentations	1 h per week	
Meeting of paramedics	1 h per month	
Staff meetings	12 h per year	
Continuous medical education	24 h per year	
Outreaches	20 days/year	
Additional activities (Group 3 activities)		
Staff supervision	12 h per year	
Supervision of students	16 h per year	
General administration	30 min per day	
Surveillance reporting	32 h per year	
Monthly reports	12 h per year	
Medical legal duties	8 h/year	

Dental therapists					
Health service/group 1	No. per year			Service standard/unit	Standard workload
Dental examinations	340			15 min/patient	8000
Referrals	20			10 min/patient	12,000
Tooth extractions	340			45 min/patient	2667
Temporary dressings	18			30 min/patient	4000
Patient management	340			45 min/patient	2667
2. Laboratory assistant					
Malaria smear	3261			25 min/sample	4800
Malaria RDT	515			10 min/sample	12,000
White cell count	3154			12 min/sample	10,000
WBC differential	194			28 min/sample	4286
ESR	146			15 min/sample	8000
Platelet count	17			12 min/sample	10,000
Total lymphocyte count	19			12 min/sample	10,000
VDRL	353			10 min/sample	12,000
TPHA	438			10 min/sample	12,000
HIV testing	182			20 min/sample	6000
Hepatitis B	242			20 min/sample	6000
Widal test	46			20 min/sample	6000
Pregnancy test	69			5 min/sample	24,000
Urinalysis strip	396			5 min/sample	24,000
Urinalysis microscopy	136			10 min/sample	12,000
Blood fluid split	17			5 min/sample	24,000
Blood fluid microscopy	15			10 min/sample	12,000
AFB	854			35 min/sample	3429
Other body fluids	36			10 min/sample	12,000
Gram stain	32			10 min/sample	12,000
Blood grouping	719			10 min/sample	12,000
Cross match	250			15 min/sample	8000
Blood collection	173			20 min/sample	6000
BSL	79			7 min/sample	17,143
Stool for OCP	10			8 min/sample	15,000
CSF exam	1			9 min/sample	13,333
Alkaline phosphate	57			6 min/sample	20,000
Amylase	14			6 min/sample	20,000
Cholinesterase	6			6 min/sample	20,000
Gamma glutamyl	58			6 min/sample	20,000
Aspartate A	75			6 min/sample	20,000
Alanine Amino	63			6 min/sample	20,000
Leucine Amino	5			6 min/sample	20,000
Albumin	61			6 min/sample	20,000
Blood urea nitrogen	81			6 min/sample	20,000
Calcium	7			6 min/sample	20,000
Creatine	116			6 min/sample	20,000
Direct bilirubin	10			6 min/sample	20,000
Total bilirubin	49			6 min/sample	20,000
Total cholesterol	1			6 min/sample	20,000
Triglyceride	9			6 min/sample	20,000
Total protein	37			6 min/sample	20,000
Uric acid	81			6 min/sample	20,000
Sodium	99			6 min/sample	20,000
Potassium	159			6 min/sample	20,000
Chloride	74			6 min/sample	20,000
C-reactive protein	4			6 min/sample	20,000
Hepatitis c	25			20 min/sample	6000
3.Health Extension Officer					
TB consultations	160			30 min/patient	3680
Deaths	2			10 min/patient	11,040
Patient reviews	160			120 min/patient	920
Admission	160			20 min/patient	5520
In patient care for TB	160			20 min/patient	5520
Ward rounds	1443			20 min/patient	5520
Dispensing of TB drugs	160			7 min/patient	15,771
Referrals	2			10 min/patient	11,040
Discharges	156			20 min/patient	5520
Assist in minor surgeries	401			17 min/patient	6494
4. Medical Officer					
Consultations	1,784			20 min/patient	5880
Admissions	1143			30 min/patient	3920
Ward rounds	1143			20 min/patient	5880
Minor surgical procedures	401			17 min/patient	6918
Intermediate surgeries	319			45 min/patient	2613
Major surgeries	70			259 min/patient	454
Medical procedures	82			30 min/patient	3920
Complex deliveries	31			60 min/patient	1960
Referrals	251			15 min/patient	7840
Assault and injuries	136			30 min/patient	3920
Death declaration	38			10 min/patient	11,760
Discharge	1401			10 min/patient	11,760
Re-attendances	7853			15 min/patient	7840
5. Midwives					
Admissions	335			20 min/patient	5664
Nursing care	335			600 min/patient	189
Labour management	335			240 min/patient	472
Ward rounds	335			20 min/patient	5664
Normal deliveries	335			45 min/patient	2517
Immediate Newborn care	362			35 min/patient	3237
Newborn care	364			15 min/patient	7552
Post- natal care	362			20 min/patient	5664
Discharges	364			10 min/patient	11,328
Care of the dead	2			30 min/patient	3776
6. Nurses—outpatient					
Antenatal clinic	352			40 min/patient	2820
Subsequent visit	420			25 min/patient	4512
Immunization injections	6176			25 min/patient	4512
Immunization oral	2348			15 min/patient	7520
Family planning—condoms	8			10 min/patient	11,280
Family planning—injection	309			20 min/patient	5640
Family planning—insertible	156			30 min/patient	3760
Family planning—natural	15			25 min/patient	4512
Family planning —oral	21			10 min/patient	11,280
Removal of implants	2108			25 min/patient	4512
Malnutrition management	240			25 min/patient	4512
Well baby clinic	240			25 min/patient	4512
7. Ophthalmic assistant					
OPD consultations	5284			20 min/patient	5448
Review of patients	796			10 min/patient	10,896
Assessment of refractions	579			25 min/patient	4358
Glass prescriptions	579			5 min/patient	21,792
Assistant in cataract surgery	182			20 min/patient	5448
Assessment of cataracts	182			35 min/patient	3113
Referrals	104			15 min/patient	7264
Removal of foreign objects	16			10 min/patient	10,896
Fundoscopy	16			15 min/patient	7264
Eye irrigation	16			20 min/patient	5448
Application of ointment	5284			10 min/patient	10,896
Post-cataract counselling	220			25 min/patient	4358
8. Nurse inpatient					
Management of TB	160			25 min/patient	4685
Care of the dead	39			35 min/patient	3346
Medical procedures	2866			45 min/patient	2603
Discharges	2866			20 min/patient	6856
Formula preparation	120			50 min/patient	2342
Burns	5			75 min/patient	1562
Post-operative care	763			35 min/patient	3346
Referrals	141			30 min/patient	3904
Wound dressing	20			74 min/patient	1583
Blood transfusion	35			30 min/patient	3904
Pre-operative care	763			45 min/patient	2603
Ward rounds	2866			75 min/patient	1562
OPD assessment	5928			20 min/patient	5856
Admissions	2866			20 min/patient	5856
Administration of medications	5928			25 min/patient	4685
Review of patients	4650			20 min/patient	5856
Sample collection	1076			20 min/patient	5856
Nursing care	2866			600 min/inpatient day	195
9. Theatre nurse					
Minor surgical procedures	384			17 min/patient	6635
Intermediate surgeries	309			45 min/patient	2507
Major surgeries	70			259 min/patient	436
10. Anaesthetic Technical Officer					
Minor surgical procedures	384			17 min/patient	7059
Intermediate surgeries	309			45 min/patient	2667
Major surgeries	70			259 min/patient	463
11. Physiotherapist					
Assessment of patients	123			60 min/patient	1760
Review of patients—wards	232			45 min/patient	2347
Ward rounds	232			10 min/patient	10,560
Management of adults	208			50 min/patient	2112
Management of minors	27			35 min/patient	3017
Manual interventions	345			74 min/patient	1427
Mobility training	180			60 min/patient	1760
Deep breathing exercises	24			45 min/patient	2347
12. Pharmacy assistant					
Dispensing of dangerous drugs	471			30 min/patient	3920
Medicines dispensing	7313			25 min/patient	4704
Pre-packaging services	7784			17 min/patient	6918
Compounding	363			40 min/patient	2940
13. X-ray assistant					
Plain radiographs	3105			30 min/patient	4000
ECG	62			45 min/patient	2667

The workload components, activity standards and the annual statistics were first uploaded in the WISN software to provide staffing requirements for health services also known as workload group 1. This was followed by uploading the support activities or workload group 2 activities and the category allowances to get the category allowance factor in percentage and a staff requirement for the support activities. Finally, additional activities or workload group 3 activities were uploaded into the software with the relevant additional allowances to provide the individual allowance standards in hours per year and the individual allowance factor which is the staff required undertaking additional activities. Using the WISN formula for calculating staff requirements of 1 × 2 + 3 = staff requirements, the staffing requirement per cadre were calculated as summarized in Table 3.

**Table 3:** WISN-calculated staffing requirements clinical staff at Braun District Hospital

Type of staff	Existing staff	Calculated requirement	Difference	WISN ratio
Dental therapists	1	1	0	1.00
Medical laboratory assistants	2	3	− 1	0.67
Health extension officers	3	1	2	3.00
Medical officers	1	4	− 3	0.25
Midwives	6	6	0	1.00
Outpatient nurses	5	4	1	1.25
Ophthalmic assistants	1	3	− 2	0.33
In patient nurses	21	33	− 12	0.64
Theatre nurses	4	1	3	4.00
Anaesthetic technical staff	2	1	1	2.00
Physiotherapists	1	1	0	1.00
Pharmacy assistants	2	4	− 2	0.50
X-ray assistants	1	2	− 1	0.50

Table 3 shows mixed results of balances, shortages and cases of staff surpluses. Balances are recorded among the midwives, physiotherapist and the dental therapist. The existing staff equal the WISN-calculated staffing requirements hence recording a difference of 0 and a WISN ratio of 1.00.

Staffing shortages were recorded across 6 cadres. The staff differences with a negative sign mean a gap or a shortage. The medical laboratory assistants have 2 existing staff and the staffing requirement calculated by WISN is 3 giving a difference of -1 and WISN ratio of 0.67. The medical officers have an existing staff of 1 and the calculated requirement based on workloads is 4 thus a difference of at -3 and WISN ratio of 0.25. The ophthalmic assistants have an existing staff of 1 and the WISN requirement estimates a total of 3 staff thus a gap of − 2 and WISN ratio of 0.33. Also, the existing inpatient nurses are 21 and the estimated WISN-calculated requirements is 33 showing a difference of − 12 and WISN ratio of 0.64. Braun Hospital currently has 2 pharmacy staff in the facility but based on the workload, the WISN estimates a requirement of 4 giving a difference of − 2 and a corresponding WISN ratio of 0.50. Finally, only 1 X-ray assistant exists in the facility and the WISN requirements estimates a total of 2 staff thus a difference of − 1 with a WISN ratio of 0.50.

There were circumstances where calculated WISN staff requirements were less than existing staff. Such were recorded by the health extension officers, theatre nurses and anaesthetic technical staff (ATO). The existing health extension officers in the facility at the time of study were 3. The WISN-calculated requirements were estimated at 1 with a WISN ratio of 3.00 hence an extra staff of + 2. On the other hand, the theatre nurse in Braun hospital were 4 and the calculated WISN requirement was estimated as 1 based on the workloads with a WISN ratio of 4.00 meaning a cadre surplus of + 3 while the ATO existing staff were 2 and the calculated requirement was 1 showing a surplus of + 1 with a WISN ratio of 2.00.

There were other results also gathered that are beneficial to the upgraded hospital from the WISN study. During the field visit to the facility, it was observed that the unit had no physiotherapy equipment, the laboratory and the pharmacy would run out of stock of needed reagents, commodities and medication required in the facility. All these have an impact on the specific cadre workload. It was also noted that most of the staff assistants like the medical laboratory assistants, ophthalmic assistant, X-ray assistant and pharmacy assistants were all community health workers (CHWs). This is an indication of staff sharing and shifting tasks where the CHW undertakes roles that are not their traditional roles.

Non-clinical staffing requirements were also estimated based on the shifting programmes. The facility had a category of non-clinical staff working for 5 days, 8 h per day with non-working days during public holidays. Such positions have 2080 h per year. The calculated staffing requirements for such posts estimated a requirement of 0.96 staff rounded up to 1. On the other hand, there were positions that require staff throughout the year for 24 h daily, 7 days a week and 365 days. They operated in 2 shifts with a total of 8736 h. Such positions require a total of 4.2 staff (8736/2080) for shifting. In other words, 4 full-time staff working in shifts is recommended for such positions like of the security officers and the drivers. This covers the continuous shift work all through the year. It was noted some non-clinical health workers engaged in more than one role. For example, the statistician functions as the electrician and a driver at the same time.

## Discussion

Upgrading a health facility requires evidence to provide the health managers with the right information to make decisions on staffing requirements and even necessary equipment to respond to the new status of the facility. Thus, the WISN results provide the staffing requirements for each of the cadres in the health facility. Some indicated shortages, other balances while others an indication of more staff than required. These results provide further insights to the health workforce decisions.

For those cadres that reported a difference of 0 and WISN ratio of 1, the results were interpreted as having a staff balance meaning that the existing staff were just sufficient to offer the services of that facility within the professional standards of the country. Thus, no action was required but sustenance of the services. However, if the new status of the facility anticipates increased workloads, the results can still be used to estimate future staffing based on expected increase of services as reported by other studies [11, 12].

For those cadres that had results showing negative differences, it was interpreted as staff shortages that require prioritization or reassignment of available cadres in cases of scarce resources like it is the situation in PNG. If the facility has funds, staff hiring can be prioritized too. For example, in the case of inpatient nurses that registered a gap of − 12, the immediate decision administrative decision before staff hiring could be to reassign one of the surplus outpatient nurses to join the inpatient nurses and reduce the workload pressure of the inpatient nurses. Alternatively, assign some of the theatre nurses to support the inpatient nurses during low seasons or when no surgical procedure cases are booked. For the medical officer, a shortage of -3 staff was registered. The immediate action could be to assign the + 2 surplus health extension officers to support in consulting at the outpatient department and only to allow the only medical officer to attend to more critical cases that require his expertise and reduce the high workload pressure.

The higher the shortages, the higher the workload pressures and shown by the ratios that are less than 1. It further means that the cadres could be working under pressure, thus services have possibilities of being compromised. On the contrary, the staff cadres whose WISN ratios were more than 1 and positive differences, it meant that the cadres in existence were more than what the facility required. There were also cases of surplus staff meaning the calculated estimated requirement was less than the existing staff. Positive differences indicate that the number of staff for specific cadre in the facility is more than the staff required to cope with the existing workload. It also means that the WISN ratios are more than 1, an indication of low workload pressures. The higher the surpluses in the differences, the higher the WISN ratio registered. Ultimately, the lower the workload pressure on the particular staff cadre. It could also imply that the quality of the services in that facility should be better than those with negative differences due to the existence of more staff. The existing staff should therefore offer the best services as they have no work pressure at all.

The results provide information that resonates with the National Health Services Standards that require health facilities to have the right staff, right equipment and technology. Level 4 health facilities or district hospitals are required to deliver medical, child health/paediatric, maternal and minor surgical services (including public health activities). They also provide clinical support services in pharmacy, pathology, anaesthetics and radiology [13]. Human resources for health play the most significant role in delivering all planned health services in any facility and thus become a key priority to policy makers [14]. It is therefore necessary that they are well sourced, equipped and managed for them to effectively function. They must be in their right numbers, skill mix, right attitudes, skills and competencies and working in the right environment [15–17] to achieve the right health targets for the projected indicators.

Our study findings strengthen similar studies that show inequities in staff distribution even within facilities [18]. We provide evidence on further uses of WISN results in making management decisions such as changing the status or levels of care based on evidence. Our study was conducted to support the LHS administration to plan for the human resources for health requirements for the upgraded hospital from a health centre.

Other results exhibit similar characteristics that have been documented in other countries where CHWs are used to undertake roles that are not their core cadres and tasks are informally shifted and shared without policies to ensure its rationality. This is mainly due to staff shortages [19, 20]. The need for formalizing task shifting/sharing in situations of scarcity such as where medical officers, nursing officers and midwives are scarce is critical. The need for a task shifting and sharing policy with guidelines to ensure quality of services is important. Capacity building of these staff should be provided and continuous supervision to ensure tasks for the new roles are effectively provided is emphasized [20, 21].

The results provide evidence that calls upon the health managers in PNG to review the role of community health workers in the health facilities. The work they undertake in the facility is far beyond their training and they tend to be easily translated into any cadre where need arises. Moreover, Braun District hospital is located in an island with no health facilities nearby thus the need for releasing the community health workers to undertake their core roles at the household levels with promotive, preventive and rehabilitative services as necessary.

Multitasking was reported among the non-clinical staff. Multitasking can have detrimental effects on task performance and increase errors. Conducting more than one task together may affect performance and increase cost in terms of decreased accuracy [22, 23] and increased reaction time to environmental stimuli [24]. Experimental studies have typically investigated this by presenting two tasks in close proximity and observing the participant’s response to both. Some of the tasks do not require full-time staff but rather outsourcing when such services are needed like the electrician and the painter. These are not activities to be conducted on daily basis. Likewise, for clinical service, multitasking has effects on the quality and time taken to conduct the task. Increasing the time between the first and the second task reduces the delay in responding to the second task. Voluntarily or internally prompted, multitasking has different implications for efficiency and errors than when externally prompted [25].

There is no doubt that the WISN tool provides a scientific method of calculating staff workloads. However, there were limitations encountered during the study. These included under recording of service statistics, the eNHIS does not capture all the tasks provided by the experts but only reports on the key indicators identified for various programmes. This was mitigated by visiting the health facility to collect raw data from the daily registers. Some of the statistics conducted by similar cadres were aggregated and it was a challenge to apportion service statistics to specific cadres and thus a percentage was used.

## Conclusion

The results from this WISN study provide evidence for basing various staffing decisions on. Some of the decisions include staff reassignment; some with surplus could mean anticipation of more clients considering the facility change of status as well staff prioritization for hiring. WISN method is an important methodology to help managers make important decisions such as changing the status of a facility like in this study; from a health centre to a district hospital.

## Data Availability

Data and materials are available on request from NDoH and LHS report.

## Abbreviations

AWT: Available working time
CHWs: Community health workers
HEO: Health extension officer
PNG: Papua New Guinea
eNHIS: Electronic National Health Information systems
WISN: Workload indicators of staffing need
LHS: Lutheran health services
